# m^6^A Methylation Mediates the Function of the circRNA-08436/miR-195/ELOVL6 Axis in Regards to Lipid Metabolism in Dairy Goat Mammary Glands

**DOI:** 10.3390/ani14121715

**Published:** 2024-06-07

**Authors:** Yu Wang, Yanni Wu, Sitian Yang, Rui Gao, Xiaoyang Lv, Zhangping Yang, Peixin Jiao, Ning Zhang, Juan J. Loor, Zhi Chen

**Affiliations:** 1 Key Laboratory of Animal Biotechnology of Xinjiang, Institute of Biotechnology, Xinjiang Academy of Animal Science, Urumqi 830000, China; 221902118@stu.yzu.edu.cn (Y.W.); xjzhangning@126.com (N.Z.); 2 Key Laboratory of Genetics Breeding and Reproduction of Grass Feeding Livestock, Minisitry of Agriculture and Rural Affairs, Institute of Biotechnology, Xinjiang Academy of Animal Science, Urumqi 830000, China; 3 College of Animal Science and Technology, Yangzhou University, Yangzhou 225009, China; mx120220877@stu.yzu.edu.cn (Y.W.); 221902425@stu.yzu.edu.cn (S.Y.); mx120230881@stu.yzu.edu.cn (R.G.); dx120170085@yzu.edu.cn (X.L.); yzp@yzu.edu.cn (Z.Y.); 4 Joint International Research Laboratory of Agriculture & Agri-Product Safety, Ministry of Education, Yangzhou University, Yangzhou 225009, China; 5 International Joint Research Laboratory in Universities of Jiangsu Province of China for Domestic Animal Germplasm Resources and Genetic Improvement, Yangzhou 225009, China; 6 College of Animal Science and Technology, Northeast Agricultural University, Harbin 150030, China; peixin.jiao@neau.edu.cn; 7 Mammalian Nutrition Physiology Genomics, Department of Animal Sciences and Division of Nutritional Sciences, University of Illinois, Urbana, IL 61801, USA

**Keywords:** circRNA-08436, miR-195, ELOVL6, M^6^A methylation

## Abstract

**Simple Summary:**

The synthesis and secretion of milk fat is a multifaceted biological process, prompting extensive research into the regulatory mechanisms governing milk fat production in regards to genetics and dairy science. Analyzing the regulation of milk fat metabolism in dairy goats provides insights for improving goat milk quality via molecular breeding strategies. Therefore, this research aims to design a series of molecular biological experiments to elucidate the molecular mechanism underlying the regulation of breast milk fat metabolism by the m^6^A methylation mediated circRNA-08436/miR-195/ELOVL6 axis. The research findings of this study will enrich the existing theories regarding fatty acid metabolism in the mammary gland tissue of dairy goats, shed new light on the production of high-quality goat milk, and provide new ways to improve the dietary structure of residents.

**Abstract:**

The nutritional value of goat milk is determined by the composition of its fatty acids, with particular importance placed on the role of unsaturated fatty acids in promoting human health. CircRNAs have been known to affect fatty acid metabolism through different pathways. In this study, high-throughput sequencing was employed to construct expression profiles of mammary tissue harvested during the dry period and peak lactation stages of dairy goats. Differentially expressed circRNAs and mRNAs were screened, revealing significantly higher expression levels of circRNA-08436 and *ELOVL6* during the peak lactation period compared with the dry period. Thus, circRNA-08436 and *ELOVL6* were chosen for subsequent studies. The findings demonstrated that circRNA-08436 not only promotes the synthesis of triglyceride (TAG) and cholesterol in goat mammary epithelial cells (GMECs), but also increases the concentrations of saturated fatty acids in the cells. Through the utilization of software prediction, the dual luciferase reporter system, and qRT-PCR, it was observed that circRNA-08436 binds to miR-195, with its overexpression reducing the expression levels of miR-195 and inhibiting TAG synthesis. In addition, circRNA-08436 upregulated the expression levels of the miR-195 target gene *ELOVL6*. The data also revealed that *YTHDC1* facilitated the transport of circRNA-08436 from the nucleus to the cytoplasm, while *YTHDC2* in the cytoplasm functioned as a “reader” to identify and degrade circRNA-08436. Taken together, these findings contribute to a better understanding of the molecular regulation of fatty acid metabolism in the mammary glands of dairy goats, thus offering a sound theoretical basis for the production of high-quality goat milk.

## 1. Introduction

Dairy goats are an important breed of livestock, with its primary product, goat milk, possessing a high nutritional value and exhibiting preventive and protective functions against various human diseases [1,2]. These functions are closely attributed to the composition and content of fatty acids in milk, with n-3, n-6, and n-9 unsaturated fatty acids and other polyunsaturated fatty acids, such as arachidonic acid and docosahexaenoic acid, playing a crucial role [3,4]. Thus, an in-depth investigation of the regulatory mechanism of fatty acid metabolism in the mammary glands of dairy goats can serve as an experimental method for improving the quality of goat milk, while also providing a reference for improving the human diet.

The regulation of fatty acid metabolism in the mammary gland involves the expression of multiple genes, including circular RNAs, as well as network regulation and signal transduction [5,6]. Ongoing research has revealed that the molecular mechanisms regulating fatty acid metabolism in the mammary gland are considerably more complex than currently understood, with numerous regulators yet to be identified and characterized. The discovery of circRNAs also presents a novel avenue for investigating the molecular mechanism of lipid metabolism, which holds substantial significance in the quest to improve milk quality [7,8].

Chen et al. reported that circ09863 promoted the synthesis of triglyceride (TAG) and increased the levels of C16:1 and C18:1 in goat mammary epithelial cells (GMECs) [9]. However, existing reports lack comprehensive explorations into the specific pathways and mechanisms through which circRNAs regulate fatty acid composition. Because of this, it is imperative to conduct a thorough study of the functional and mechanistic aspects of circRNAs in fatty acid metabolism within the mammary gland. Such investigations will facilitate the effective utilization of circRNAs for regulating the fatty acid composition of goat milk, thereby offering practical value for the development of the dairy goat industry.

The role of N6-methyladenosine (m^6^A) in the modification of mRNA molecules is well known [10,11]. The presence of m^6^A in the mRNAs of mammalian cells was first discovered in the 1970s, but its function and mechanism of action have received limited attention. Methylation modification of m^6^A is now emerging as a prominent hotspot for RNA epigenetics research in the field of livestock and poultry genetics [12]. There is, however, little information regarding the mechanism by which m^6^A regulates milk lipids produced by the mammary gland in dairy goats. Accordingly, this study explores the molecular mechanisms through which circRNA-08436 exerts its function in the context of methylation modifications that occur after the initiation of lactation in dairy goats.

## 2. Materials and Methods

### 2.1. Ethics Statement

The animal use and care protocol was approved by the Animal Use and Care Committee of Yangzhou University (202203089), Yangzhou, China. The Saanen goats used in this study were obtained from the experimental farm of Yangzhou University. All Saanen goats, three years old and undergoing their second lactation, were subjected to the same feeding conditions and were provided with free feeding and access to clean water. The sampling process was divided into two distinct time points, i.e., dry period (-7 DIM, days in milk) and peak lactation (180 DIM, days in milk). From each time point, three Saanen goats with similar body weights were selected for blood collection and euthanasia.

### 2.2. Experimental Animals and Preparation of Samples

Mammary gland tissues were surgically collected from goats in the two periods (dry period (−7 DIM, days in milk) and peak lactation (180 DIM, days in milk)). Three samples were pooled and snap-frozen immediately in liquid nitrogen. Total RNA was extracted using Trizol reagent (Invitrogen, Carlsbad, CA, USA), according to the manufacturer’s instructions. The quantity and quality of RNA were determined using a Nano Drop ND-1000 spectrophotometer (NanoDrop, Waltham, MA, USA), and the RNA was stored at −80 °C before use.

### 2.3. Differential Expression Analysis of circRNAs

Total RNA was extracted with Trizol reagent, according to manufacturer’s instructions. The mass and concentration of total RNA were assessed via the Bioanalyzer 2100 and RNA 6000 Nano LabChip Kit (Agilent, CA, USA). All libraries were quality checked and sequenced using Illumina Hiseq 2000/2500 (Illumina, San Diego, CA, USA), with a sequencing read length of two *150 bp (PE150). All raw reads generated by high-throughput sequencing were saved in the FASTQ format to obtain high-quality reads that could be used for subsequent analysis. The functional annotation of circRNAs was determined by their formation mechanism, while their Gene Ontology (GO) annotation and their Kyoto Encyclopedia of Genes and Genomes (KEGG) annotation were mainly based on the function of the corresponding circRNA-hosting genes. Based on the grouping of samples and the FPKM value of circRNA expression, the DEseq software package was employed to analyze the differential expression of circRNAs. The circRNAs were considered to be differentially expressed with a *T*-test result of *p* < 0.05 and log^2^ fold change ≥ 2.

The raw readings generated by high-throughput sequencing were saved in the FASTQ format to obtain reliable high-quality readings suitable for subsequent analysis. The comparison results between the net reading and the reference genome were stored in binary files, commonly referred to as BAM files. The FPKM value of the genes was quantified using Cufflinks, while the readings of each sample were analyzed using HTSeq count software to calculate the expression differences in the genes. The data were subsequently standardized using DESeq R software, enabling the calculation of *p* values (<0.05) and fold-changes (≥two times) of the observed differences.

### 2.4. Cell Culture and Treatment

As for GMEC separation, we collected mammary glands of milk goats in the peak lactation period. Then, we used 3× PBS to rinse them and placed them into 3× double anti-d-Hanks solution (Pricella Corp, Wuhan, China). Next, we maintained the glands at a lower temperature and took them back to the laboratory, quickly placing them in 3× PBS, cutting the acinar, removing the connective tissues, and cutting the tissue into pieces, holding the samples in a small dish at 37 °C for 30 min. Finally, we retrieved these tissues and added 1 mL F12 medium to them, changing the medium after 2 days. The basal medium for the primary mammary epithelial cells of Saanen goats consisted of DMEM/F12 (Invitrogen, Carlsbad, CA, USA) and 10% fetal bovine serum (Sigma, Kansas City, MO, USA), supplemented with various cytokines (10 ng/mL EGF-1 (epidermal growth factor 1, Gibco), 5 mg/mL insulin, and 0.25 mmol/L hydrocortisone). The separated mammary epithelial cells were cultured at 37 °C, 5% CO_2_, under suitable humidity. The cells were observed every 48 h, and the culture medium was replaced. To induce lactogenesis, GMECs were cultured in a lactogenic medium (including 2 μg/mL of prolactin) for 24 h prior to the initial experiments. Cells were cultured and transfected with small RNAs or other interventions using Lipofectamine™ RNAiMAX (Invitrogen, Carlsbad, CA, USA), according to the manufacturer’s instructions. Cells were subjected to the fractionation of the nucleus and cytoplasm using the Cytoplastic & Nuclear RNA Purification Kit (Norgen Biotek, Richmond, VA, USA).

### 2.5. Determination of TAG and Cholesterol

The cells were cultured in six-well plates until they reached full confluence and achieved a suitable density for experimentation. Generally, the cell status was observed after 48 h of treatment. Following a thorough rinse with PBS, the PBS was removed, and 150 μL of lysate was added. The mixture was then incubated at 4 °C for 15 min before collecting the cells and lysate into 1.5 mL EP tubes. The cells were subsequently disrupted on ice, and the supernatant was collected through centrifugation. The working solution was configured for subsequent processing (Loogen, Beijing, China). After this, 10 μL of sample solution was obtained for BCA protein assay (Prod#23227, Thermo Corp, Waltham, MA, USA). The resulting values were used to determine the protein concentration and were calibrated using the BCA protein values.

### 2.6. Determination of Intracellular Fatty Acid Content

Intracellular fatty acids were extracted for methyl esterification, according to the methods described in previous studies [13]. Briefly, 100 mg of the cells was added to two mL of 0.25% methanol sulfate solution. The fatty acids underwent methyl esterification via an ultrasonic crushing treatment and incubation at 80 °C for 1 h. After the solution was cooled to room temperature, 800 μL of n-hexane was added, and the mixture was subjected to vortex shaking for 30 s to ensure proper mixing. The resulting solution was then allowed to cool to room temperature. Centrifugation was performed at 900× *g* for 5 min at room temperature. The resulting supernatant was then transferred to a centrifuge tube made of siliconized glass. Approximately 0.5 g of anhydrous sodium sulfate was added to the tube, followed by vigorous vortex shaking to achieve dehydration. After centrifugation at 13,800× *g* for 5 min at room temperature, the supernatant was aspirated. Gas chromatography–mass spectrometry (GC–MS) (Agilent CrossLab, Beijing, China) was conducted on the aspirated supernatant in a PEG-20M column to determine the fatty acid content.

### 2.7. Oil Red O Staining

For the oil red O staining experiment, a six-well plate was utilized. The 10% paraformaldehyde solution was directly added to the culture plate, which was then discarded after 40 min. The plate was cleansed with PBS three times. The cells were washed once with 60% isopropanol, cleaned with PBS, and allowed to dry at room temperature. Next, staining was performed by adding 1 mL of 5% oil red O solution, which was discarded after 1 h. The cells were then washed with PBS solution more than three times. The changes in oil red O were observed under a microscope.

### 2.8. 5-Ethynyl-2′-Deoxyuridine (EdU) Experiment

The GMECs were inoculated into a six-well culture plate, which was replaced by culture medium for 12 h when cell growth reached 80% confluency. The EdU solution (Thermo Fisher, Waltham, MA, USA) was diluted with Opti-MEM medium in a ratio of 1000:1 and added to the cells, along with 50 μL of PBS containing 4% paraformaldehyde. After fixing the cells at room temperature for 30 min, Apollo staining and DNA staining were performed, and the images were collected for analysis.

### 2.9. Quantitative Real-Time Polymerase Chain Reaction (qRT-PCR)

The extracted RNA was subjected to RNase R treatment at 37 °C for 15 min. The cDNA synthesis was performed using TaKaRa’s PrimeScript RT regent Kit reverse transcription kit (TaKaRa, Dalian, China). For this purpose, a universal reverse transcription system was prepared, consisting of total RNA (500 ng), 5× Mix (2 μL), random 6-base length primer (0.5 μL), oligo DT primer (0.5 μL), and ddH_2_O to make up a final volume of 10 μL. The reverse transcription program involved treatment at 37 °C for 15 min, brief treatment at 85 °C for 5 s, and final storage at 4 °C. The real-time PCR (Thermo Fisher, Waltham, MA, USA) was carried out using a Bio-Rad CFX96 instrument (Hercules, CA, USA). The PCR reaction system consisted of SYBR Prmix Ex Taq II (12.5 μL), cDNA (10 ng), and upstream and downstream primers (10 μmol/L) (Table S1), and the final reaction volume was 25 μL, with replenished ddH_2_O. Relative expression was calculated by 2^−ΔΔCT^, with β-actin as an internal reference.

### 2.10. Construction of Circ08436 Overexpression Vector

The full-length sequence of circRNA was amplified, and the primer was designed with restriction endonuclease *HindIII* and *KpnⅠ* cleavage sites and protection bases at both ends. Subsequently, the full-length sequence of circ08436, containing loop-forming junction sequences, was ligated to the pcDNA3.1 vector. For the construction of the dual luciferase reporter vector, primers were designed to amplify the circ08436 sequence containing the miR-195 binding site, with restriction endonuclease *NotⅠ* and *XhoⅠ* cleavage sites and protective bases at both ends. This amplified sequence was then ligated into the psiCHECK-2 vector.

### 2.11. Western Blotting

Total cellular proteins were extracted, and 20 µg was used for electrophoresis. After separation, the proteins were transferred onto a PVDF membrane using a semi-dry transmembrane apparatus and then blocked with 5% skim milk at room temperature for two hours. The membrane was then incubated with the primary antibody overnight at 4 °C. After washing five times with TBST for 5 min each, the membrane was incubated with the secondary antibody at room temperature for 2 h. Next, the membrane was again washed five times with TBST. The protein signals were detected using a chemiluminescent ECL protein blotting system (Pierce, Waltham, MA, USA). ELOVL6 (elongase of very long chain fatty acids 6) expression was detected employing the ELOVL6 rabbit anti-bovine polyclonal antibody (21160-1-AP, Proteintech Corp, Wuhan, China) as the primary antibody and sheep anti-rabbit IgG-HRP (Tiangen, Beijing, China) as the secondary antibody. Expression of the internal reference β-actin was detected using the β-actin mouse anti-bovine monoclonal antibody (66009-1-IG, ProteintechGronup, Wuhan, China) as the primary antibody and sheep anti-mouse IgG-HRP as the secondary antibody (Tiangen, Beijing, China). BCA kits (Thermo, Beijing, China) were used for protein quantification.

### 2.12. Dual-Luciferase Activity Analysis

The 293A cells were digested and inoculated into 48-well plates at a density of approximately 7 × 10^4^ cells per well, and their status and density were evaluated 24 h later. When they were fully adherent and reached 75% confluence, the reporter plasmid vectors were transfected with PEI transfection reagent (Gobekie, Shanghai, China) in three replicates per group. After four hours, the fluorescence was observed via fluorescence microscopy. After 48 h, the cells were washed with PBS three times. Subsequently, 40 μL of 1 × Passive Lysis Buffer was added, and the cells were then shaken on a shaker for 15 min. Next, the collected liquid was centrifuged in a 1.5 mL EP tube for 10 min at ‘3500’× rpm. A total of 4 μL of supernatant was mixed with 20 μL of LARII, followed by fluorescence detection using Lumat3 LB9508 (Roche, Basel, Switzerland). Subsequently, 20 μL of termination solution was added, and the fluorescence values of the internal reference were recorded during the test. The test data were subjected to analysis, normalization, and processing to generate the fluorescence report test results. The binding sites between circ00-8436 and miR-195, as well as between miR-195 and ELOVL6, were predicted using the CircInteractome database and the TargetScan database, respectively (Table S2). HEK293T cells were cotransfected with pcDNA-miR-195 and recombinant psiCHECK-2-circ08436-W/psiCHECK-2-circ08436-Mut (pCK-circ08436-W/pCK-circ08436-Mut) vectors, and the targeting relationship was verified using a dual luciferase gene reporter vector assay (Promega, Beijing, China).

### 2.13. Statistical Analysis

In this study, the gene expression data were analyzed by *t*-tests using SPSS 19.0 (SPSS, Inc., Chicago, IL, USA). The luciferase activity data and the gene expression data were analyzed by one-way ANOVAs using the above SPSS system. Statistical differences were considered significant at *p* value < 0.05 and highly significant at *p* value < 0.01. GraphPad Prism 9. 0 was used for graph generation.

## 3. Results

### 3.1. High-Throughput Sequencing of circRNAs from Mammary Gland Tissue of Dairy Goats

**Figure 1:**
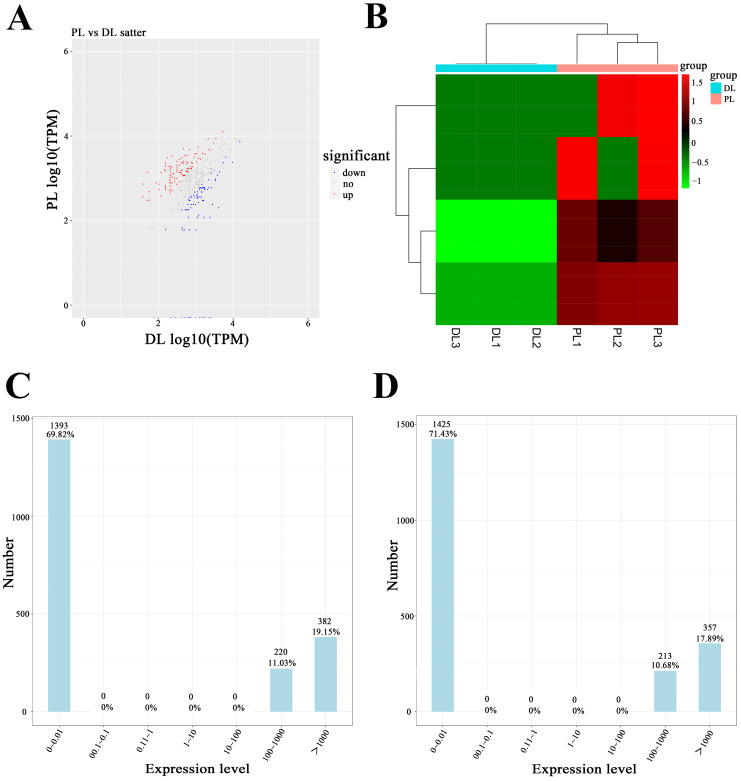
Differential expressions of circRNAs in mammary tissues of Saanen goats during the dry period and peak lactation. (**A**): Volcano plot of circRNAs expressed differently between the dry period and peak lactation; (**B**): heatmap of circRNAs expressed differently between early lactation and peak lactation; (**C**): TPM distribution of dry period; (**D**): TPM distribution of peak lactation.

The analysis identified 64 circRNAs with a multiplicity of difference with fold change >2 and *p* value < 0.05. We identified 174 differentially expressed circRNAs between dry-period and peak-lactation mammary tissue, with 103 upregulated and 71 downregulated (Figure 1, Table S3). In addition, the expression level of circRNA-08436 was upgraded by 5.11-fold.

### 3.2. Differential Expression Analysis of mRNAs

**Figure 2:**
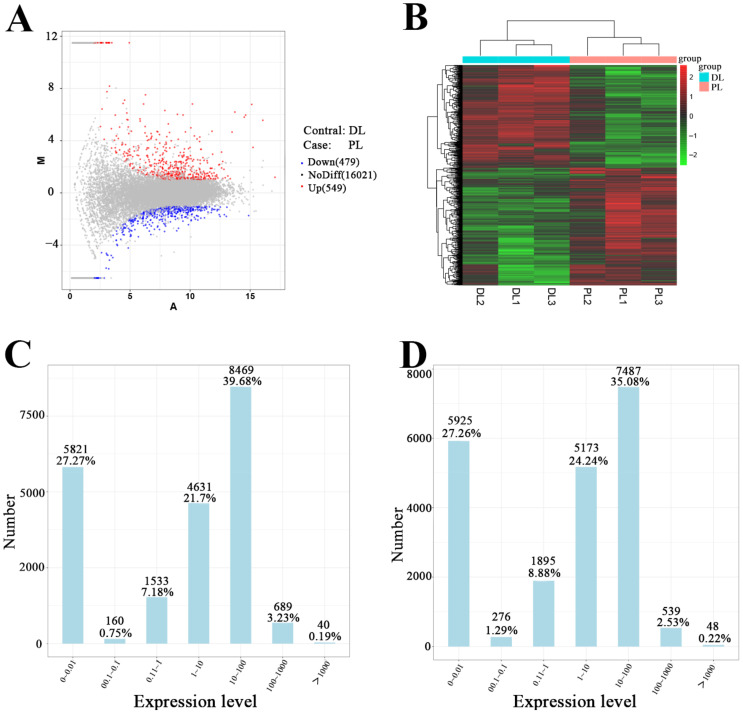
Differential expression of mRNAs in breast tissues of Saanen goats in the dry period and at peak lactation. (**A**): Volcano plot of mRNAs expressed differently between dry period and peak lactation; (**B**): heatmap of mRNAs expressed differently between early lactation and peak lactation; (**C**): FPKM distribution in dry period; (**D**): FPKM distribution in peak lactation.

A total of 3096 mRNAs were found to be statistically significant, with a *p* value < 0.05 and a fold change >2. Among these mRNAs, 402 were upregulated, and 394 were downregulated. These identified mRNAs hold potential as research targets (Figure 2). In addition, the expression of *ELOVL6* was upregulated by a fold change of 2.07 (Table S4). Notably, differences in the expression of *ABCG1* (ATP Binding Cassette Subfamily G Member 1), *ABCA1* (ATP Binding Cassette Subfamily A Member 1), *CD36* (cluster of differentiation), and *ACACA* (Acetyl-CoA Carboxylase Alpha), all of which were related to fatty acid metabolism, were also observed, thus, validating the accuracy of the sequencing results.

### 3.3. Enrichment Analysis and Pathway Network Diagram of Differential Genes

**Figure 3:**
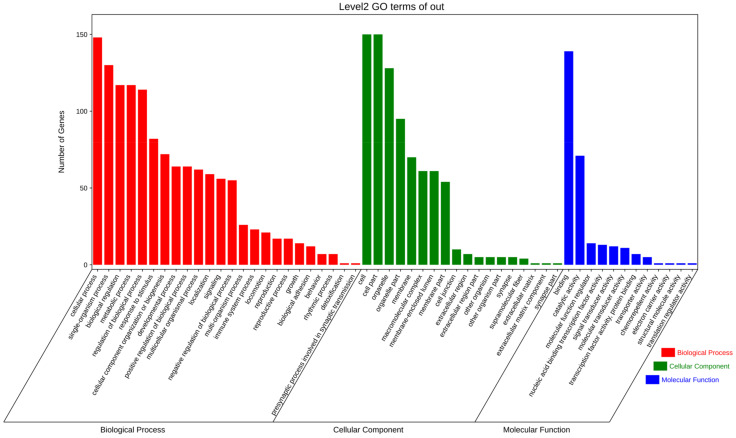
GO enrichment analysis.

**Figure 4:**
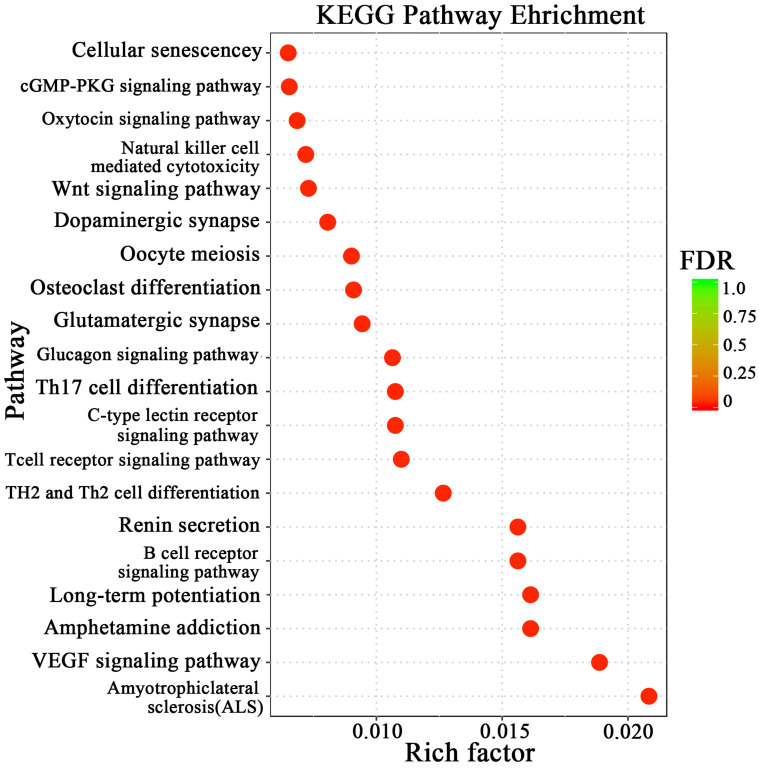
KEGG enrichment analysis.

The GO enrichment analysis revealed 54 categories of differentially expressed circRNAs, including molecular function, biological process, and molecular function regulator (Figure 3). Additionally, KEGG analysis identified 61 enriched pathways, including cellular senescence, the Wnt signaling pathway, glutamatergic synapse, the T cell receptor signaling pathway, etc. (Figure 4). These results indicate that the circRNAs produced by these genes may play multiple roles in the mammary gland through these pathways.

The GO enrichment analysis results of differentially expressed circRNA source genes were classified into GO categories based on molecular function, biological process, and molecular function regulator.

According to the KEGG enrichment results, the degree of enrichment is measured by the Rich factor and the number of circRNA source genes enriched on this pathway. Among these, the Rich factor refers to the ratio of the number of differentially expressed circRNA source genes enriched in the pathway to the number of annotated differentially expressed circRNA source genes. The larger the Rich factor, the greater the degree of enrichment.

### 3.4. Adsorption and Binding of circRNA-08436 to miR-195

**Figure 5:**
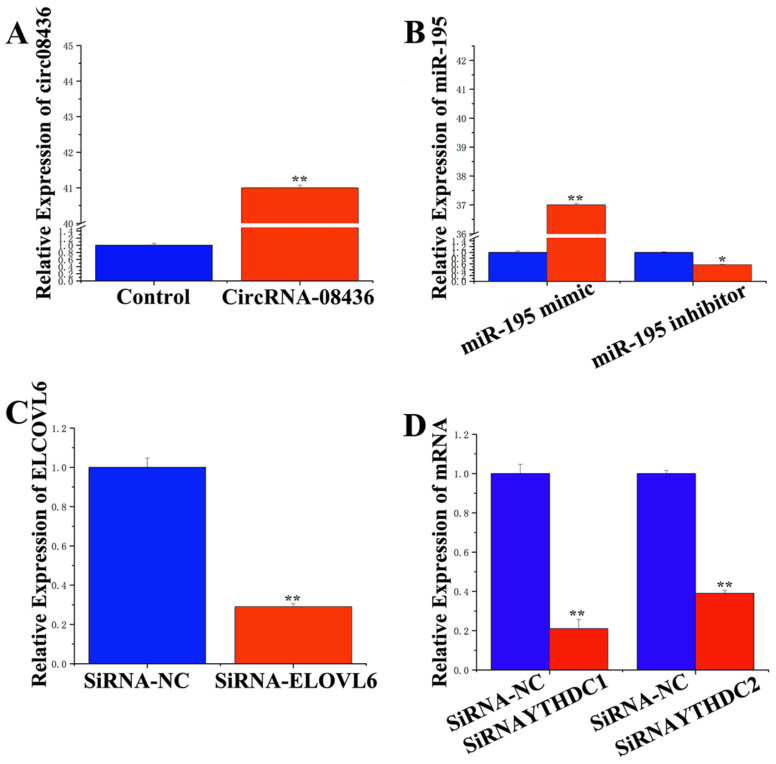
Transfection efficiency of circ08436, miR-195, siRNA-ELOVL6, and siRNA-YTHDC1/2. (**A**): The expression efficiency of circ08436 overexpression vector pcDNA-circ08436; qRT-PCR quantification of circ08436 expression (n = 6). Blue bars represent negative control; red bars represent circ08436. (**B**): The expression level of miR195; qRT-PCR quantification of miR-195 expression (n = 6). Blue bars represent negative control; red bars represent miR-195 mimic or inhibitor. (**C**) The mRNA expression level of *ELOVL6*; qRT-PCR quantification of *ELOVL6* expression (n = 6). Blue bars represent the siRNA-NC; red bars represent the siRNA-ELOVL6. (**D**) The mRNA expression level of *YTHDC1* and *YTHDC2*; qRT-PCR quantification of *YTHDC1* and *YTHDC2* expression (n = 6). Blue bars represent the siRNA-NC; red bars represent the siRNA-*YTHDC1* and siRNA-*YTHDC2*. * *p* < 0.05; ** *p* < 0.01.

We constructed a circ08436 sequence hyperexpression vector (pcDNA-circ08436, Tables S5 and S6) and assessed its expression efficiency in GMECs using qRT-PCR (Tables S5 and S6). Our findings revealed a significant 41-fold increase in the expression of circ08436 (Figure 5A), indicating its suitability as a super-expression vector for subsequent studies. After treatment, the expression of miR-195 mimic in GMECs exhibited a 37-fold upregulation compared to that of the NC-mimic group (Figure 5B). Conversely, the expression of the miR-195 inhibitor decreased by 59% compared with that of the NC-inhibitor group. Furthermore, the expression of siRNA-ELOVL6 in GMECs decreased by more than 70% compared with that of the NC-siRNA group (Figure 5C).

**Figure 6:**
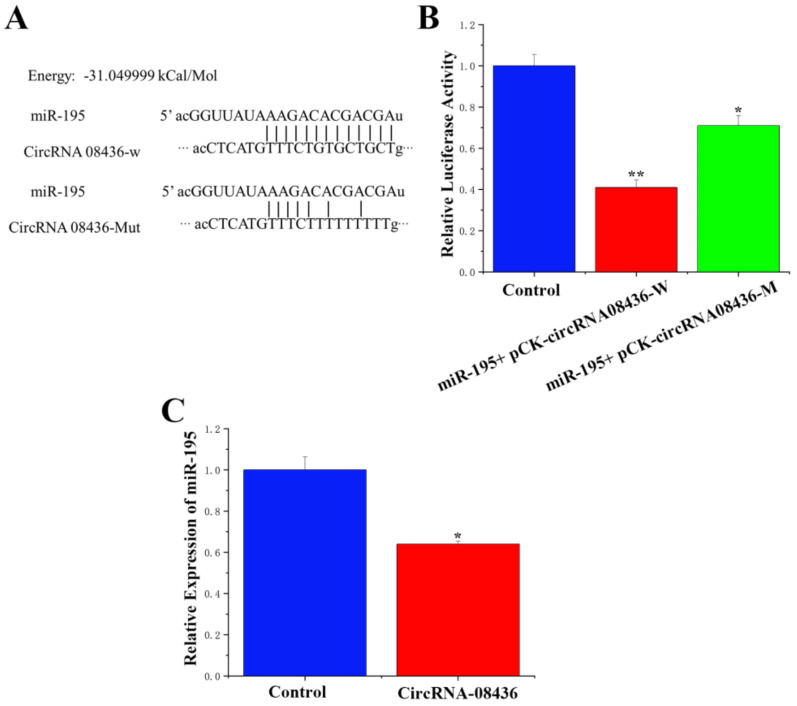
Analysis of the relationship between circRNA-08436 and miR-195. (**A**): CircRNA-08436 and miR-195 binding site analysis; (**B**): fluorescence activity analysis of HEK293T cells after co-transfection of miR-195 and pCK-circRNA-08436-W/pCK-circRNA-08436-Mut; (**C**): the effect of circRNA-08436 on the expression level of miR-195; qRT-PCR quantification of miR-195 expression (n = 6). Blue bars represent the negative control; red bars represent circ08436. * *p* < 0.05; ** *p* < 0.01.

Furthermore, sequence analysis demonstrated the presence of a binding site between the circRNA-08436 and miR-195 sequences (Figure 6A). To confirm the binding ability of circRNA-08436 to miR-195, we employed PCR to recombine the sequence containing the binding site of miR-195 into the psiCHECK 2 vector, resulting in the formation of a wild-type recombinant vector. The mutant psiCHECK-2 vector was generated by mutating the binding site through the overlapping PCR method. The luciferase reporter gene assay, which assessed the activity expression of both wild-type and mutant luciferase, revealed a significant decrease in the activity of luciferase after the co-transfection of the wild-type vector and miR-195. Conversely, there was no significant change in luciferase activity after co-transfection of the mutant vector and miR-195 compared with that of the control group (Figure 6B). Furthermore, the presence of circRNA-08436 significantly reduced the expression levels of miR-195 (Figure 6C).

### 3.5. Specific Targeting of miR-195 on ELOVL6

**Figure 7:**
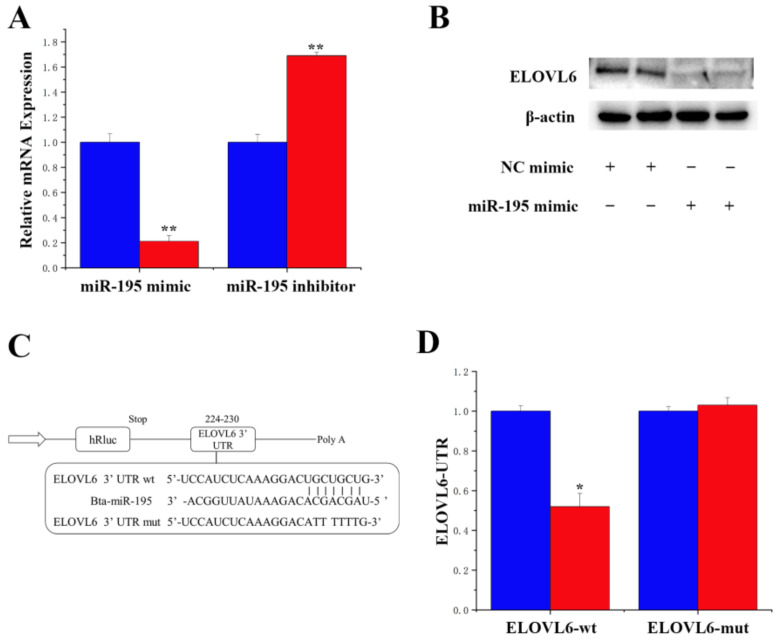
Specific targeting of miR-195 on ELOVL6. (**A**): The mRNA expression level of ELOVL6 in the miR-195 mimic and miR-195 inhibitor, respectively. Blue bars represent negative control; red bars represent miR-195 mimic or inhibitor. (**B**): The protein expression level of ELOVL6. The effect of miR-195 mimics and inhibitor ELOVL6 protein expression was evaluated by Western blot analysis of the cell. (**C**): Target site of miR-195 in ELOVL6 3′-UTR. (**D**): The construction of the luciferase (Luc) vector fuses with the ELOVL6 3′-UTR. WT: Luc reporter vector, with the WT ELOVL6 3′-UTR (34 to 40); MUT: Luc reporter vector, with the mutation at miR-195 site in ELOVL6 3′-UTR. * *p* < 0.05; ** *p* < 0.01.

DAVID and TargetScan analyses results indicated that miR-195 is one of the miRNAs that binds to the 3′-UTR of ELOVL6. Subsequently, we detected ELOVL6’s mRNAs and protein expression levels, which presented downregulated expression upon the overexpression of miR-195, while demonstrating upregulated expression upon the inhibition of miR-195 (Figure 7A,B). To provide further evidence of the direct targeting of ELOVL6 by miR-195, we conducted a series of experiments. We first synthesized a 3′-UTR fragment containing ELOVL6, the miR-195 targeting site. This fragment was then cloned into the psi-CHECK2 vector, resulting in the construction of a 3′-UTR plasmid. Subsequently, the luciferase report detection was performed to assess the impact of the overexpression of miR-195 on the luciferase activity of the wild-type and mutated reporter genes. The results demonstrated a reduction in luciferase activity in the wild-type reporter gene 3′-UTR. In contrast, no significant change was observed in the luciferase activity of the mutated reporter gene (Figure 7C,D). These findings indicated that miR-195 directly targets the 3′-UTR site of ELOVL6, exerting a negative regulatory effect.

### 3.6. Functional Verification of circRNA-08436 in GMECs

**Figure 8:**
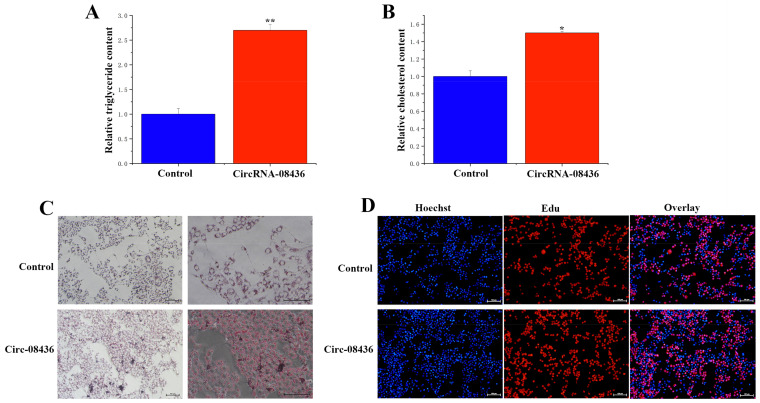
Functional verification of circ08436 in GMECs. (**A**): TAG levels in cells transfected with circ08436. Blue bars represent the negative control; red bars represent circ08436. (**B**): Cholesterol relative levels. Blue bars represent negative control; red bars represent circ08436. * *p* < 0.05; ** *p* < 0.01. (**C**): Oil red O staining observation results of blank group and circ08436 group in GMECs. (**D**): EdU observation results of blank group and circ08436 group in GMECs.

**Figure 9:**
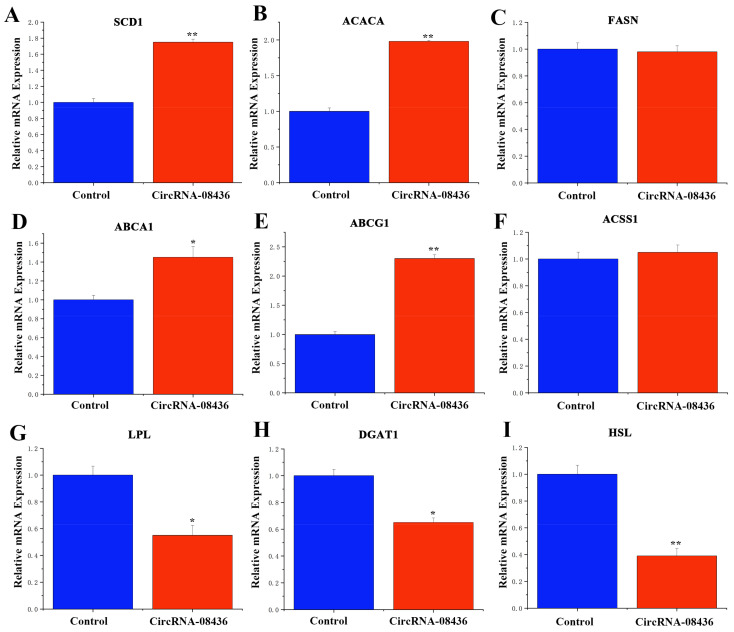
Functional verification of circ08436 in GMECs. (**A**–**I**): Effects of circ08436 on genes associated with lipid deposition and secretion in GMECs. The mRNA expression of *SCD1*, *ACACA*, *FASN*, *ABCA1*, *ABCG1*, *ACSS1*, *LPL*, *DGAT1*, and HSL was quantified by qRT-PCR (n = 6). Blue bars represent the negative control; red bars represent circ08436. * *p* < 0.05; ** *p* < 0.01.

Following the overexpression of circ08436, there was an increase in the TAG levels of GMECs (Figure 8A), as well as in cholesterol levels and lipid droplet secretion (Figure 8B,C). These changes were accompanied by increases in the content of saturated fatty acids (C16:1 and C18:1) in the cells (Table 1), while no significant differences were detected in regards to cell differentiation (Figure 8D). Moreover, the overexpression of circ08436 significantly increased the expression of *SCD1*, *ACACA*, *ABCA1*, and *ABCG1*, whereas it had no significant effect on the expression of *FASN* (Fatty Acid Synthase) and *ACSS1* (Acyl-CoA Synthetase Short Chain Family Member 1). Furthermore, *LPL* (Lipoprotein lipase), *DGAT1* (Diacylglycerol O-Acyltransferase 1) and *HSL* (Homo sapiens) were significantly downregulated (Figure 9).

### 3.7. Functional Role of miR-195 and ELOVL6 in GMECs

**Figure 10:**
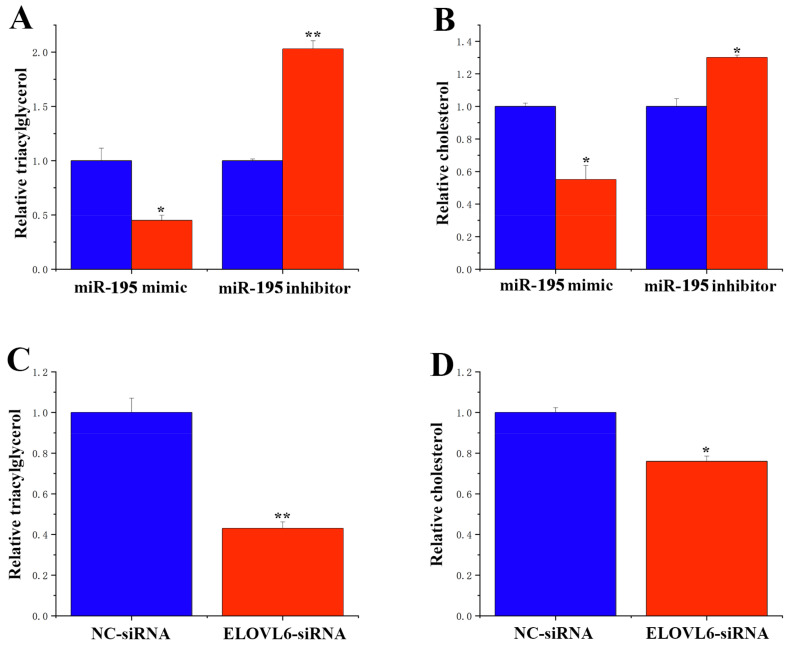
Functional evaluation of miR-195 and ELOVL6. (**A**): TAG relative levels. TAG levels in cells transfected with the miR-195 mimic or inhibitor. Blue bars represent the negative control; red bars represent the miR-195 mimic or inhibitor. (**B**): Cholesterol relative levels. Cholesterol levels in cells transfected with the miR-195 mimic or inhibitor. Blue bars represent negative control; red bars represent miR-195 mimic or inhibitor. (**C**): TAG relative levels. TAG levels in cells transfected with ELOVL6. Blue bars represent the negative control; red bars represent the siRNA-ELOVL6. (**D**): Cholesterol relative levels. Blue bars represent negative control; red bars represent siRNA-ELOVL6. * *p* < 0.05; ** *p* < 0.01.

To verify the specific function of miR-195 in GMECs, we measured TAG and cholesterol levels in GMECs upon miR-195 overexpression or inhibition. Our results revealed that miR-195 exhibited a significant inhibitory effect on TAG content, reducing it by more than 50%. Conversely, inhibition of miR-195 led to an increase in TAG content (Figure 10A). Furthermore, its overexpression resulted in a significant reduction in cellular cholesterol content (Figure 10B). Further functional exploration of the miR-195 target gene *ELOVL6* demonstrated that siRNA-*ELOVL6* significantly reduced both TAG (Figure 10C) and cholesterol content (Figure 10D).

### 3.8. Combination of circ08436 with miR-195 to Relieve the Inhibition of ELOVL6

**Figure 11:**
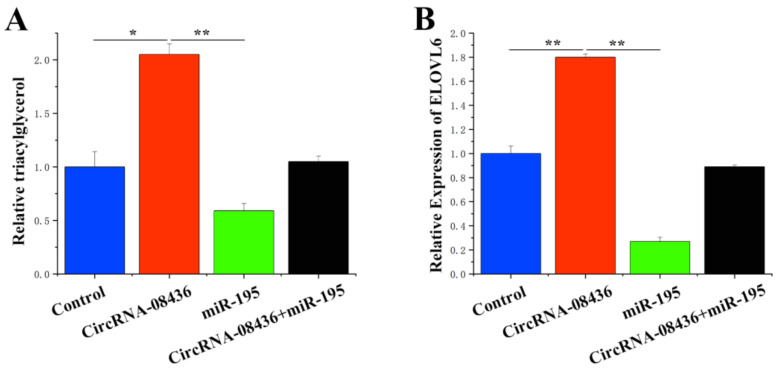
Combination of circ08436 with miR-195 to relieve ELOVL6 inhibition. (**A**): TAG levels in cells transfected with control, circ08436, or circ08436 + miR-195. (**B**): Circ08436 promotes mRNA expression of ELOVL6; qRT-PCR quantification of ELOVL6 expression (n = 6). ELOVL6 expression levels in cells transfected with control, miR-195, circ08436, and Circ08436 + miR-195. * *p* < 0.05; ** *p* < 0.01.

To substantiate the functional regulatory relationship between circ08436 and miR-195/*ELOVL6*, a “remediation” experiment was conducted. Figure 11A depicts the positive effect of circ08436 on the TAG content in the GMECs. However, when miR-195 and circ08436 were treated simultaneously in the GMECs, the effect of circ08436 on TAG was diminished. In addition, miR-195 was observed to target *ELOVL6* to inhibit the expression of *ELOVL6*, whereas circ08436 promoted the expression of *ELOVL6*. However, when both circ08436 and miR-195 were jointly transferred into the GMECs, the positive effect of circ08436 on *ELOVL6* was neutralized by miR-195 (Figure 11B).

### 3.9. Regulation of circRNA-08436 by m^6^A Methylation

**Figure 12:**
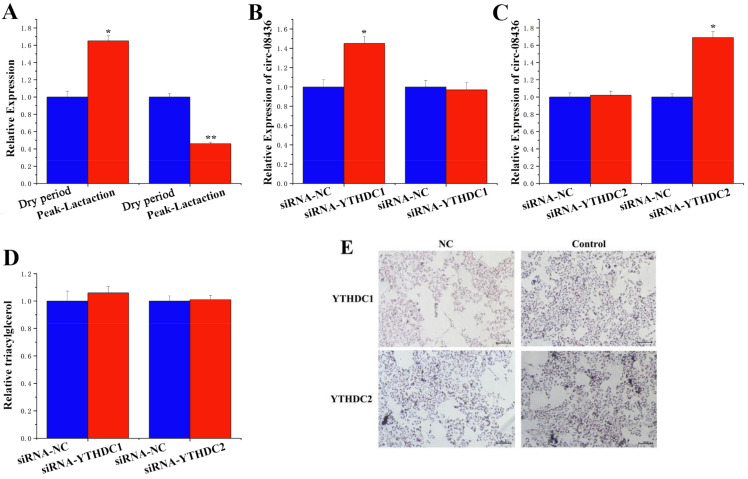
Regulation of circRNA-08436 by m^6^A methylation modification. (**A**): YTHDC1 and YTHDC2 expression level in the dry period and the peak lactation; qRT-PCR quantification of YTHDC1 and YTHDC2 expression (n = 6). Blue bars represent the dry period; red bars represent peak lactation. (**B**): YTHDC1 expression level in the cytoplasm and nucleus; qRT-PCR quantification of circ08436 expression (n = 6). Blue bars represent the siRNA-NC; red bars represent the siRNA-YTHDC1. (**C**): YTHDC2 expression level in the cytoplasm and nucleus; qRT-PCR quantification of circ08436 expression (n = 6). Blue bars represent the siRNA-NC; red bars represent the siRNA-YTHDC2. (**D**): TAG relative levels of siRNA-YTHDC1 and siRNA- YTHDC2; blue bars represent the siRNA-NC; red bars represent the siRNA-YTHDC1 or siRNA-YTHDC2. (**E**): Oil red O staining observation of siRNA-YTHDC1 and siRNA-YTHDC2. * *p* < 0.05; ** *p* < 0.01.

High-throughput sequencing revealed a significant upregulation of YTH N6-Methyladenosine RNA Binding Protein C1 (*YTHDC1)* expression at the peak lactation stage compared with that of the dry period. In contrast, the expression of YTH N6-Methyladenosine RNA Binding Protein C2 (*YTHDC2)* was significantly downregulated during both the dry period and peak lactation (Table S4). The accuracy of these sequencing results was further validated through quantitative analysis (Figure 12A). In addition, the inhibition of *YTHDC1* resulted in a substantial increase in circRNA-08436 levels in the nucleus, while there was no significant difference in its expression in the cell cytoplasm (Figure 12B). the inhibition of *YTHDC2* did not result in a notable alteration in circRNA-08436 expression in the cell nucleus, while a noticeable increase in its expression in the cytoplasm was observed (Figure 12C). Lipid metabolism, as demonstrated by TAG and oil red O staining, was unrelated to *YTHDC1* and *YTHDC2* (Figure 5D and Figure 12D,E).

## 4. Discussion

### 4.1. CircRNA-08436 Can Promote TAG Synthesis

The circRNA was first discovered in plants in 1976. However, its complete validation was impeded by technical limitations until the substantial advancement of high-throughput sequencing technology [14]. In 2012, researchers used RNA-Seq to identify circRNAs in different human cells, marking its official inclusion in the scientific domain and garnering remarkable attention [15,16,17]. Its discovery has not only opened a new avenue for investigating the molecular mechanism of fatty acid metabolism in the mammary gland, but also holds crucial significance for improving milk quality. Few studies have been conducted on the regulation of lipid metabolism by circRNAs alone. Following screening of differentially expressed circRNAs through KEGG analysis, circRNA-08436, whose expression was upregulated during the peak lactation stage, was selected as the research subject. The function of circRNA-08436 in GMECs was validated through various assays, including TAG and cholesterol content detection, oil red O and EdU detection, and qPCR analysis of genes related to milk fat synthesis. The findings indicated that circRNA-08436 can promote TAG synthesis at the peak lactation stage.

### 4.2. CircRNA-08436 Can Competitively Bind to miR-195, Thereby Deregulating the Inhibitory Effect of miR-195 on the Target Gene ELOVL6

Numerous studies have revealed that circRNAs function as molecular sponges for microRNAs (miRNAs), possessing one or even multiple binding sites for these molecules. This interaction hinders the binding of miRNAs to their target genes by adsorbing specific miRNAs binding sites, consequently impeding the functionality of miRNAs. Some studies have reported that circRNAs inhibit the regulation of fatty acids by binding miRNAs to their target genes through the absorption of specific miRNAs binding sites [18,19,20]. This mechanism has been extensively studied in the regulation of milk fat synthesis in dairy cows. Previous studies have demonstrated that peroxisome proliferator-activated receptor γ (*PPARγ*) and peroxisome proliferator-activated receptors α (*PPARα*) can induce Achaete-Scute Family BHLH Transcription Factor 1 (*ASCL1*) gene expression [21] leading to an increase in long-chain fatty acid content [22]. As reported by Chen et al., circ09863 can promote the synthesis of TAG in GMECs and increase concentrations of C16:1 and C18:1 [9]. Consistent with these results, our study hypothesized that circRNA-08436 also functions as a miRNA sponge. It was predicted that miR-195 would possess a binding site with circRNA-08436, which led to the construction of a wild-type vector for circRNA-08436 and subsequent co-transfection with miR-195. These findings indicated that circRNA-08436 is capable of adsorbing miR-195, thereby reducing its expression level.

Given that miRNAs exert their biological functions by inhibiting the translation of target mRNAs or by promoting the degradation of mRNAs through binding to the 3′ UTR [23], we used an online miRNA target bioinformatics prediction database to identify potential targets of miR-195. *ELOVL6* was subsequently validated as a target gene. This gene belongs to the family of ultra-long-chain fatty acid elongases (ELOVLs) and is responsible for catalyzing the elongation of saturated and monounsaturated fatty acids [24]. Its primary functions involve fatty acid elongation and lipoyl CoA biosynthesis [25,26]. The studies on the *ELOVL6* gene have mainly focused on fatty acid metabolism, oxidative stress, inflammation, and other metabolic diseases [27,28,29]. Despite its importance as a regulatory gene in caprine fatty acid metabolism [30], there are few mechanistic studies of the role of *ELOVL6*. The fact that knocking down *ELOVL6* results in a significant decrease in TAG and cholesterol content indicated that this gene plays a crucial role in promoting milk lipid synthesis in GMECs. Through the “remediation” assay, we verified that circRNA-08436 can competitively bind to miR-195, thereby deregulating the inhibitory effect of miR-195 on the target gene *ELOVL6*. This interaction subsequently regulates the functions of TAGs and fatty acids.

### 4.3. YTHDC2 May Play a Role in the Degradation of circRNA-08436 in the Cytoplasm

The process of m^6^A RNA modification involves methyltransferases, demethyltransferases, and reading proteins (readers). Most of these readers exhibit unique mechanisms for different biological functions. For instance, *YTHDF2* is the first ever reported reader protein identified to mediate the degradation of target mRNAs. *YTHDF1* not only enhances the translation of m^6^A-modified mRNAs but also influences the overall translational output [31]. *YTHDF3* promotes both the translation and degradation of mRNAs, and *YTHDC1* assumes a key role in many biological functions and disease progressions [32,33]. CircRNAs are commonly considered to be byproducts of co-transcription, resulting from the splicing of typical linear mRNAs and primarily occurring in the nucleus. However, studies have increasingly revealed that a substantial proportion of circRNAs are located in the cytoplasm, indicating the criticality of investigating the mechanism underlying the export of circRNA from the nucleus to the cytoplasm [9,15]. The function of m^6^A modification is mediated by the m^6^A selective “reading” protein belonging to the YTH family, which binds m^6^A-modified mRNAs, thereby influencing RNA metabolic pathways [34]. In a study conducted in 2017, the m^6^A reading protein *YTHDC1* was found to play a crucial role in the transportation of m^6^A methylated mRNAs from the nucleus to the cytoplasm [35]. Chen et al. further verified this idea by revealing that *YTHDC1*, a member of the YTH structural domain proteins, binds to circNSUN2, thereby facilitating the action of splicing factors and assisting in the export of circNSUN2 from the nucleus to the cytoplasm [36]. These findings highlighted the significance of *YTHDC1* as a key factor in the transport of circRNAs from the nucleus to the cytoplasm under m^6^A modification.

In another study assessing the interaction between YTHDF and circRNAs, it was determined that, although circRNAs are widely recognizable by the YTHDF family, their stability is diminished under the regulation of *YTHN6*-methyladenosine RNA binding protein 2 (*YTHDF2*) [37]. The experiments of Zaccara et al. explained this phenomenon, attributing it to the dissimilar structure of *YTHDF2* in comparison to those of other YTH families, suggesting that *YTHDF2* serves additional functions beyond its primary role of translation enhancement [38]. Compared with the dry period, *YTHDC1* was significantly upregulated during peak lactation, while the expression of *YTHDC2* was exactly the opposite. In addition, upon inhibition of *YTHDC1*, circRNA-08436 levels in the nucleus significantly increased, while no salient disparity in circRNA-08436 expression was observed in the cytoplasm. These data suggested that the export of circRNA-08436 from the nucleus to the cytoplasm is contingent upon the involvement of *YTHDC1*. Under overexpression of *YTHDC2*, there was no remarkable difference in circRNA-08436 expression in the nucleus, while a significant reduction in its expression was observed in the cytoplasm. This finding suggested that *YTHDC2* may play a role in the degradation of circRNA-08436 in the cytoplasm, but neither this nor *YTHDC1* affect the content of TAGs and lipid droplets in GMECs.

## 5. Conclusions

**Figure 13:**
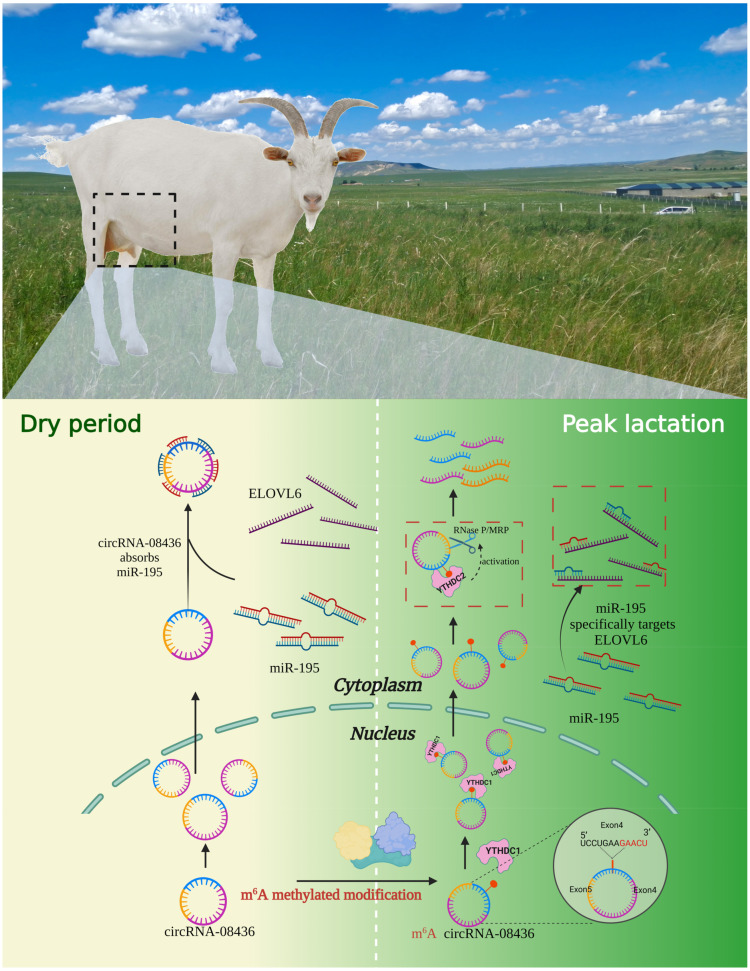
M^6^A methylation mediates the molecular mechanism of circRNA-08436/miR-195/ELOVL6 axis regulation in dairy goat milk metabolism.

This study has identified that following lactation in goats, circRNA-08436 undergoes m6A methylation modification. YTHDC1 promotes the transport of circRNA-08436 from the nucleus to the cytoplasm, where it binds to miR-195 to release miR-195 specific targeted ELOVL6. The undisturbed ELOVL6 further promotes the generation of unsaturated fatty acids (Figure 13). These findings significantly contribute to the establishment of a theoretical and empirical foundation for comprehending the molecular mechanism whereby circRNAs regulate lipid metabolism.

## Figures and Tables

**Table 1:** Effect of circ08436 on fatty acid composition in GMECs.

Fatty Acid	Control	circ08436
C16:0 (%)	21.41 ± 0.14	41.12 ± 0.56 **
C16:1 (%)	10.12 ± 0.57	2.10 ± 0.34 **
C18:0 (%)	10.57 ± 0.34	18.41 ± 0.25 *
C18:1 (%)	48.14 ± 0.35	34.83 ± 0.41 **
C18:2 (%)	9.76 ± 0.24	3.54 ± 0.24 *
SFA (%)	31.98	59.53
UFA (%)	68.02	40.47
UFA/SFA	2.13	0.68

Note: SFA, saturated fatty acid; UFA, unsaturated fatty acid. Relative fatty acid composition is calculated as a percentage relative to the total fatty acids identified. All experiments were performed in duplicate and repeated six times. Comparison between control and circ08436, * *p* < 0.05; ** *p* < 0.01.

## Data Availability

Data are contained within the article and Supplementary Materials.

## Supplementary Materials

The following are available online at https://www.mdpi.com/article/10.3390/ani14121715/s1, Table S1: Primer sequence of mRNA; Table S2: Dual-luciferase activity sequence; Table S3: High-Throughput Sequencing of circRNAs; Table S4: High-Throughput Sequencing of mRNAs; Table S5: Sequence of circRNA-08436; Table S6: Primer sequence of circRNA-08436.
